# The prevalence of disordered eating in elite male and female soccer players

**DOI:** 10.1007/s40519-020-00872-0

**Published:** 2020-02-27

**Authors:** Will Abbott, Adam Brett, Thomas E. Brownlee, Kelly M. Hammond, Liam D. Harper, Robert J. Naughton, Liam Anderson, Edward H. Munson, Jack V. Sharkey, Rebecca K. Randell, Tom Clifford

**Affiliations:** 1 Brighton and Hove Albion F.C, American Express Elite Performance Centre, Lancing, UK; 2 Research Institute for Sport and Exercise Sciences, Liverpool John Moores University, Liverpool, UK; 3 School of Human and Health Sciences, University of Huddersfield, Huddersfield, UK; 4 Crewe Alexandra Football Club, Alexandra Stadium, Gretsy Road, Crewe, Cheshire UK; 5 Harlequins Football Rugby Club, Twickenham, London, UK; 6 Aston Villa Football Club, Villa Park, Birmingham, UK; 7 Gatorade Sports Science Institute, Life Sciences R&D, PepsiCo, Leicester, UK; 8 School of Sport, Exercise and Health Sciences, Loughborough University, Loughborough, LE11 3TU UK; 9 Institute of Cellular Medicine, Newcastle University, Newcastle on Tyne, UK

**Keywords:** Mental health, Football, Eating disorders, Nutrition, Sports psychology

## Abstract

**Purpose:**

To examine the prevalence of disordered eating (DE) in elite male and female soccer players and the influence of perfectionism.

**Methods:**

Using a cross-sectional design, elite male (*n* = 137) and female (*n* = 70) soccer players and non-athlete controls (*n* = 179) completed the clinical perfectionism questionnaire (CPQ-12) and the eating attitudes test (EAT-26) to assess perfectionism and DE risk, respectively.

**Results:**

Male soccer players had higher EAT-26 scores than controls (10.4 ± 9.9 vs. 6.8 ± 6.7; *P* = 0.001), but there were no differences in the prevalence of clinical levels of DE (EAT-26 score ≥ 20) (15 vs. 5%, respectively; *X*^2^ = 0.079) The proportion of females with DE risk was higher in controls [EAT-26: 13.9 ± 11.6 (25% of population)] than female players [EAT-26: 10.0 ± 9.0% (11% of population)] (*X*^2^ = 0.001). With linear regression, perfectionism explained 20% of the variation in DE risk in males (*P* = 0.001); in females, athletic status (player vs. control) and perfectionism were significant predictors of DE risk, explaining 21% of the variation (*P* = 0.001). Male reserve team players had higher EAT-26 (+ 3.5) and perfectionism (+ 2.7) scores than first-team players (*P* < 0.05). There were no differences in the prevalence of DE risk between the male and female soccer players (*X*^2^ = 0.595).

**Conclusions:**

The prevalence of DE risk was not different in elite male and female soccer players; in fact, the prevalence was greatest in non-athlete female controls. Perfectionism is a significant predictor of DE risk in males and females.

**Level of evidence:**

III, case–control study.

## Introduction

Over the past 2 decades, a number of studies have emerged suggesting disordered eating (DE) which is more prevalent in athletes than non-athletes [1–8]. The specific reasons are not entirely clear, but a number of studies suggest that the pressure to perform, achieve the optimal body shape, the regular engagement in dieting behavior, the frequent relationship changes, or a health complication can increase the risk of DE in athletes [9, 10]. Irrespective of the etiology, DE can have significant negative consequences for an athlete’s health and performance, especially if it develops into a clinical eating disorder such as anorexia nervosa or binge eating disorder [10, 11]. Unsurprisingly, research into its prevalence, risk factors, and management remains an important topic in elite sport [12, 13].

It has been suggested that athletes who compete in sports where it is advantageous to have a lower fat mass (e.g., cycling, gymnastics, swimming, and running,) are more likely to exhibit DE than athletes from other sports [13]. Perhaps as a result, there are far fewer studies examining the prevalence of DE in sports less associated with leanness, such as soccer, hockey, basketball, and rugby, typically referred to as team or intermittent sports [14]. Nonetheless, of the few studies conducted, it is clear that team-sport players are not necessarily immune to DE. For example, in a study by Sundgot-Borgen et al. [8], 24% of the elite Norwegian soccer players studied met the criteria for an eating disorder.

Of all the team sports, soccer is by far the most popular and widely played, with the Fédération Internationale de Football Association (FIFA) estimating that over 260 million people play soccer across the globe, a number that has no doubt increased since the last count in 2006 [15]. Yet, despite the high number of players, there is very limited information on DE symptoms in soccer compared to other sports, and no data on the prevalence of DE in male players. While, generally, DE is more prevalent in females, this does not mean that DE is completely absent in males, with several studies suggesting male athletes exhibit signs of potentially concerning DE [11, 16].

The lack of studies with soccer players to date suggests that they might be viewed to be at a lower risk of DE than athletes in other sports. However, recent studies have suggested that soccer players exhibit low energy availability, which is associated with DE [17]. Furthermore, it is also important to consider that several of the risk factors associated with DE are highly relevant to professional soccer players, such as intense pressure to perform [18] and fractious relationships with coaching staff [19]. Taken together, it would seem pertinent to evaluate the prevalence of DE in elite soccer players to establish the magnitude of the problem and if regular screening is required in this sport. Thus, the primary aim of this study was to assess the prevalence of DE risk in a sample of professional male and elite female soccer players using a clinically validated screening survey. We hypothesized that elite male and female soccer players would score higher on measures of DE than non-athlete controls, and that perfectionism would be a significant predictor of DE.

## Methods

### Participants and study design

The soccer participants consisted of male and female players from clubs in the English and Scottish soccer leagues, either in the first or reserve teams. Our sample size calculation was based on findings from a previous study in college-level vs. non-college-level athletes [20]. We calculated that at 80% power, and an *α* of 0.05, to detect a 5-point (8 standard deviation points) difference in EAT-26 scores, 40 volunteers were required per group. A small proportion of the male soccer players were from the top tier of the Brazilian league (< 20%). The players were recruited through contacts at clubs in each of these leagues during the 2018–2019 pre-season period. To be included in the study players had to be ≥ 18 years old, irrespective of sex, and for the male players, they had to be a current and contracted full-time professionals. The criteria for female players differed, because many leagues are still semi-professional—we, therefore, only included players who played in the top 4 leagues in that country to ensure that they were of the highest standard. Other than age and sex, no other demographic information was collected from the participants. This was recommended to us through consultations with practitioners at the club who advised us to make the survey unidentifiable and as short as possible to increase potential engagement. The control, non-athlete sample was recruited via emails to colleagues and advertisements on social media and throughout the authors’ universities. To be included in the study, they had to be ≥ 18 years old and not playing sport professionally. Participants were asked to disclose their activity levels and anyone indicating a higher than moderate-activity levels (defined as some daily activity, e.g., walking to work, walking the dog) were excluded. The study received ethical approval from Newcastle University Faculty of Medical Sciences (ethics number: 1481/4227/2018; date granted: 04/06/2018). All participants provided informed consent prior to completing the survey. Data collected were anonymous and not identifiable to any player, club or individual.

## Measures

Two clinical questionnaires were included in the online survey distributed to the clubs; The Eating Attitudes Test (EAT-26) and The Clinical Perfectionism Questionnaire (CPQ-12) (see below for further details of both questionnaires). The EAT-26 assesses DE and served as our primary outcome measure. This questionnaire was specifically chosen because it has been shown to be valid and reliable in a variety of populations and has been used in several previous studies with athletes, allowing comparisons [4, 6, 7]. It has also been suggested that self-report measures such as the EAT-26 are useful screening tools in non-leanness focused sports such as soccer [3]. We included the CPQ-12 because secondary aim of this research was to examine potential predictors of DE in this population. We specifically chose to examine perfectionism over other psychological predictors of disordered eating (e.g., depression or self-esteem), because high levels of perfectionism have consistently been associated with DE pathology in athletes and is widely believed to be one of the most significant mediators of this disorder [21]. Ideally, we would have included additional psychological measures, but as mentioned above, after consulting with several sports medicine practitioners when designing the survey, we were advised to keep the survey short to increase compliance rates. Thus, we opted to limit the number of questionnaires in the survey and, from a psychological point of view, focused on the mediating effect of perfectionism in DE in this population.

### Eating attitude test

The EAT-26 was developed by Garner and colleagues [22] and is one of the most widely used tests of eating disorder symptomatology in clinical research settings. Participants are required to select a response from 5 options (always, usually, often, sometimes, rarely, and never) that are given a numeric value (0–5). Typical questions are “I’m terrified of being overweight” and “[I] Feel that food controls my life”. The sum of all responses is the individual’s EAT-26 score; a score of 20 or more is considered indicative of DE behavior and was used as the cut-off in the present study. The Cronbach’s alpha in the present study was 0.87.

### Perfectionism

The CPQ-12 was developed by Shafran et al. [23] and contains 12 questions relating to perfectionism. The questionnaire first defines perfectionism and then asks individuals to indicate their motivation to reach certain standards over the past month. For each of the 12 items, individuals are asked to rate how well each question describes them from ‘not at all’ to ‘all the time’. They were asked to base their answers on their standards to reach goals unrelated to eating, weight, or appearance. A typical question is “Over the past month, have you felt a failure as a person because you have not succeeded in meeting your goals?”. The CPQ-12 is widely used and has been shown to be a reliable and valid measure of perfectionism in clinical and community populations [24]. The Cronbach’s alpha in the present study was 0.75.

## Data analysis

Descriptive data are presented as means ± standard deviations unless stated otherwise. All data were checked for normality and were considered normally distributed if *P* > 0.05 on the Shapiro–Wilk test. Data not normally distributed were log transformed prior to analysis. Differences in age, EAT-26, and CPQ-12 scores between the groups were analysed with independent samples *t* tests. Chi-square statistics were used to compare the percentage of participants in the two groups scoring > 20 in the EAT-26.

The predictor variables (age, CPQ-12, and athletic status) were entered into a hierarchical linear regression, with age controlled for in the first step [25] and EAT-26 as the dependent variable. Athletic status was dummy coded (e.g., soccer player = 1 and non-athlete control = 2) for inclusion in the regression model. Data analysis was performed with SPSS for Windows (SPSS version 24.0) and statistical significance was set to *P* < 0.05.

## Results

The number of participants who took part in the study and the means and SD of the study variables are presented in Table 1. The majority of respondents played in the top 2 leagues (≥ 80%); because there were so few participants in the lower leagues, a sub-analysis of data from different leagues was not performed, and all players were grouped together.

**Table 1:** EAT-26 and CPQ-12 scores for male and female soccer players and non-athlete controls

	Male						Females					
	Soccer players			Controls			Soccer players			Controls		
	*n*	EAT-26	CPQ-12	*n*	EAT-26	CPQ-12	*n*	EAT-26	CPQ-12	*n*	EAT-26	CPQ-12
Totals	157 (100)	10.4 ± 9.9^a^	24.6 ± 5.3^a^	41 (100)	6.8 ± 6.7	22.1 ± 5.7^a^	70 (100)	10.0 ± 9.0^c^	24.7 ± 5.4	138 (100)	13.9 ± 11.6	24.2 ± 5.7
Team												
First	78 (50)	8.6 ± 7.4	23.3 ± 4.8									
Reserves	79 (50)	12.1 ± 11.6^b^	26.0 ± 5.4^b^									
EAT-26 ≥ 20*	24 (15)	29.1 ± 10.8	28.5 ± 5.5	2 (5)	32.0 ± 0.0	29.0 ± 2.8	8 (11)^c^	30.6 ± 10.2	29.3 ± 5.2	35 (25)	30.7 ± 9.1	27.9 ± 5.8

Values in parenthesis are proportion of *n* (%)

EAT-26, eating attitudes test-26; CPQ-12, clinical perfectionism questionnaire

^*^Clincial cut-off indicative of DE risk

^a^Different to male controls; ^b^different to first team; ^c^different to female controls (*P* < 0.05)

### Males

The male soccer players were younger than the controls (21 ± 5 years (range: 18–36) vs. 25 ± 6 years (range: 21–39); *P* = 0.001). The male soccer players had higher EAT-26 (P = 0.001) and CPQ-12 scores (*P* = 0.004) than the non-athlete controls (Table 1). However, athletic status did not influence the proportion of males with EAT-26 scores ≥ 20 (*X*^2^ = *P* = 0.079). In the regression model, step 1 controlled for the effects of age and this was a significant predictor of EAT-26 scores. Adding CPQ-12 and Athletic Status in Step 2 increased the predictive power of the model, but only CPQ-12 was significantly associated with EAT-26 scores (Table 2).

**Table 2:** Results for males from regression analysis of the independent predictors on the dependent variable, EAT-26

Predictor	*B*	SE (*B*)	*ß*
Step 1			
Age	− 0.937	0.271	− 0.240*
Step 2			
Age	− 0.410	0.277	− 0.105
CPQ-12	1.491	0.255	0.392*
Athletic status	− 0.055	0.065	− 0.059

Step 1 *R*^2^ = .058; Step 2 *R*^2^ 0.206 *Δ* *R*^2^ = 0.148

*P < 0.05

Because there was a significant proportion of male respondents playing in the reserve teams, we performed a sub-analysis to assess for differences between these players and found that EAT-26 and CPQ-12 scores were higher in the reserve players (*P* = 0.020 and *P* = 0.002, respectively). However, there was no difference in the proportion of players with EAT-26 scores ≥ 20 between reserve and first-team players (18% vs. 13%, respectively; *X*^2^ = *P* = 0.520). Multicollinearity between the predictor variables was considered acceptable as Variation Inflation Factors were 1.5.

### Females

Female soccer players were younger than the controls (23 ± 4 years (range: 18–32); vs. 26 ± 6 years (range: 19–40); *P* = 0.001). CPQ-12 scores did not differ between groups (*P* = 0.510), but EAT-26 scores were higher in the non-athlete controls (*P* = 0.027; Table 1). A higher proportion of female controls were also at risk for an eating disorder (EAT-26 ≥ 20) compared to the female soccer players (*X*^2^ = 0.001). In the regression analysis, CPQ-12 and athletic status, but not age, were found to be significant predictors of EAT-26 scores (*P* < 0.05; Table 3). Athletic status and the CPQ-12 accounted for 21% of the variation in EAT-26 scores. Multicollinearity was < 1.0 for all variables.

**Table 3:** Results for females from regression analysis of the independent predictors on the dependent variable, EAT-26

Predictor	*B*	SE (*B*)	*ß*
Step 1			
Age	− 0.321	0.278	0.080
Step 2			
Age	0.026	0.264	0.007
CPQ-12	1.648	0.240	0.443*
Athletic status	0.137	0.051	0.172*

Step 1 *R*^2^ = .006, Step 2 *R*^2^ = 0.218, Δ *R*^2^ = 0.212

*P < 0.05

Overall, there were no differences in EAT-26 (*P* = 0.865) or CPQ-12 (*P* = 0.978) scores between the male and female soccer players, and no differences in the proportion of players scoring ≥ 20 on the EAT-26 (*X*^2^ = 0.595).

## Discussion

The principal findings of this study are that; (1) female non-athletes were at a greater risk of developing DE than female soccer players; (2) male soccer players had higher EAT-26 scores than male non-athletes, and reserve players had higher EAT-26 and CPQ-12 scores than first-team players and; (3) perfectionism was a significant predictor of DE risk, in both males and females. This study represents the most comprehensive evaluation of the prevalence of DE risk in elite level soccer players conducted to date.

In the first study to assess DE prevalence in elite soccer players, Sundgot-Borgen and colleagues [8], using clinical interviews, found that 24% of the 69 female soccer players which they studied met the criteria for DE. The prevalence rate in their study is much higher than in the present study (~ 13%), possibly due to methodological differences (sample and measures used). In a study with similar methods to ours, in terms of screening tools used, Izquierdo et al. [26] reported similar rates of DE risk to the present study, finding that ~ 11% of male and female soccer players were at risk or were suffering from DE. In the only other study, to our knowledge, to assess the prevalence of DE risk in elite level soccer players, none of the 36 professional female soccer players taking part scored higher than the clinical cut-off of 20 on the EAT-26 [7]. By comparison, 8/70 of the female soccer players and 25/137 of the male soccer players reported scores ≥ 20 in the present study, which explains why the average EAT-26 scores in our study is much higher compared to their study (~ 10 vs. 3). The reason for the stark discrepancy between their study and ours is unclear, but it could be related to the cultural differences, as their participants were from a national league in the US, or possibly because of the comparatively lower sample size in their study. Regardless of the precise reasons, the methodological inconsistencies in the studies conducted to date and general paucity of available data make it difficult to determine the true prevalence of the DE in elite professional soccer players and highlights the need for additional research.

Counter to our hypothesis, DE risk was not greater in elite soccer players compared to non-athlete controls. This was less clear in the males, as the overall EAT-26 scores were higher in the soccer players. However, because the proportion scoring ≥ 20 was not significantly higher in the male soccer players compared to the non-athlete controls, and athletic status was not a significant predictor of EAT-26 in our regression model, the risk for developing DE was unlikely to be heightened in the male soccer players.

In females, the controls were at a greater risk for DE than the female soccer players. This was confirmed by the regression analysis, which found lower athletic status to be a significant predictor of DE risk in females. This is in contrast to the findings of Sundgot‐Borgen [8], who found that the proportion of female soccer players with eating disorders was similar to a non-athlete control sample (24% vs. 23% of the cohort). Other studies in elite soccer players did not include a control group for comparison [7, 26]. However, studies in other sports have also found DE risk to be similar or greater in female non-athletes than athletes [20, 27]. For example, in Wollenburg et al. [20], collegiate athletes playing a range of sports scored lower on the EAT-26 (6.6) than non-athlete controls (16.6). Combined with our data, these findings suggest that female athletes might be less likely to develop DE than the general population. Based on the data in the present study, it is unclear why this might be, given that perfectionism scores were similar in the two cohorts and age, although lower in the female soccer players, was not predictive of overall EAT-26 scores. This means that other factors not measured in this study are likely to explain the main differences in DE risk between the soccer players and controls. In this regard, Wollenburg et al. [20] suggested that female athletes might have better self-esteem and body image satisfaction than non-athletes, making them less susceptible to behaviors associated with DE such as strict dieting or depression. This view is counter to the dominant theory that female athletes are at a greater risk of DE than the general population, but, perhaps, this view does not extend to soccer players and is limited to athletes taking part in leanness focused sports [2].

Perfectionism is recognized as a key psychopathological trait underpinning the development of DE [23]; hence, in the present study, its measurement was prioritized over other putative DE predictors. In agreement with our hypothesis, levels of clinical perfectionism explained some of the variance in EAT-26 scores in both males and females. Although this is the first study to assess perfectionism and DE risk in elite male and female soccer players, our findings are consistent with studies in athletes from other sports, and also non-athletes, which have observed strong associations between perfectionism and DE risks [23, 28, 29]. Our findings build upon the current knowledge of DE predictors and indicate that perfectionism is an intra-personal character trait associated with the risk for DE in professional soccer players. Such perfectionist traits are likely embedded in athletes’ psyche, as obsessively striving for unrealistic standards, a key aspect of perfectionism, is arguably tantamount to elite performance [2]. However, aberrant levels of perfectionism might need to be mitigated to reduce the risk of DE. In addition, we also found that perfectionism scores were greater in the male soccer players, which could help to explain, at least in part, why EAT-26 scores were higher in the players than the controls. The effects were small, however, so further studies are needed to determine if levels of perfectionism can distinguish those at a greater risk of DE in male professional soccer players.

There were no differences in perfectionism or DE risk between the female and male soccer players. This is in contrast to the prevailing consensus that female athletes are at a greater risk for developing DE than male athletes [11–13]. However, this view is limited by the fact that far less research has been conducted with male participants [11]. Our data could be sport specific though, and merely suggest that there are no differences in the risk for developing DE between male and female soccer players. In contrast, the greater risk for DE observed in non-athlete females is wholly consistent with the observations that DE is more prevalent in females than males in the general population [30].

The proportion of those scoring ≥ 20 on the EAT-26 was no different between the reserve and first-team male soccer players (18 vs 13%, respectively). Intriguingly though, EAT-26 and clinical perfectionism scores were, on the whole, higher in the reserve team players, suggesting that the higher level or more senior players exhibited less risk for DE. This is somewhat in contrast to the previous studies that suggest higher level athletes are at a greater risk for DE than sub-elite level athletes, possibly due to greater attention and pressures to perform [4, 20]. These findings are also in contrast to Prather et al. [7], who found no differences in EAT-26 scores between sub-elite and professional female soccer players. There are many potential differences in study design and cohorts between ours and these studies that could explain the discrepant findings, most notably the different sports, level, and sex of the participants. However, our findings do raise intriguing questions specific to soccer; for instance, perhaps, reserve team players have lower levels of self-esteem or feel more pressure to perform, as they are trying to break (back) into the first-team and/or earn a new contract. It is conceivable that this could lead to greater attempts to obsessively control their performance, body shape, and diet, triggering DE [1, 3, 11, 12]. Nonetheless, because there were no differences between the reserve and first-team players in terms of clinical DE levels (e.g., EAT-26 ≥ 20), the subtle differences in average EAT-26 scores observed might not signify any meaningful increase in the overall risk for developing DE.

This study has several limitations that need to be acknowledged. First, like in another similar study [8], the non-athlete controls and soccer players were not matched for age. Despite this, we do not think that this would have influenced the results, as the regression analysis revealed that age was not a significant predictor of EAT-26 scores in females, and only had a small influence on males. The findings are also limited in that we did not include measures for several psychological risk factors for DE, including self-esteem and body satisfaction. As expressed in the methods, this was because we did not want to lengthen the survey and increase the burden on the athletes, risking poor compliance. However, we acknowledge that this limits our findings, from an etiological point of view, and encourage future studies to include measures for other risk factors for DE if possible. Finally, because our participants were predominantly from the UK, the results might not be representative of all nationalities and cultures. Notwithstanding, a key strength of this study is that we evaluated DE in professional soccer players, both male and female, and had sample sizes larger than that presented in the previous studies in this population [7, 8, 26]. Previous studies have also largely limited their analysis to sub-elite athletes and only females, meaning that their findings have limited transferability to elite male populations. Thus, despite the acknowledged limitations, this study advances our understanding of the prevalence and risk factors for DE in professional male and female soccer players.

It is also important to re-iterate that this study only assessed the risk for DE via an anonymous, self-report questionnaire. Although the EAT-26 is widely used and has good clinical sensitivity, it can only discern individuals who might be at a greater risk of developing DE, a diagnosis that requires clinical follow-up. Thus, the findings are limited to prevalence estimations of the general risk for DE within the populations as opposed to clinical diagnosis of those who suffer from an eating disorder. Therefore, future research is still required to determine how many of the soccer players who scored ≥ 20 on the EAT-26 would be diagnosed with a clinical eating disorder, and if this prevalence is greater than that of the general population.

## Conclusion

This study found that the risk for DE is much greater in female non-athletes than female soccer players. In male soccer players, DE was broadly similar to that of female soccer players and male non-athletes; however, EAT-26 and perfectionism scores were higher in male soccer players, especially those in the reserve teams. High levels of perfectionism accounted for 18–20% of the variation in DE risk in males and females, confirming the important mediating role of perfectionism in DE pathology. Future studies should include additional putative predictor measures for DE such as self-esteem and body image, and clinical interviews to better understand the true prevalence and mechanisms that increase the risk of developing DE in soccer players.

### What is already known on this subject?

Over the past 2 decades, a number of studies have emerged, suggesting that disordered eating is more prevalent in athletes than non-athletes. However, this supposition is largely based on studies in female athletes from leanness focused sports, such as swimming, gymnastics, and cycling. In the present study, we examined the prevalence of disordered eating risks in both elite male and female soccer players, to determine whether disordered eating is also prevalent in these athletic populations.

### What does this study add?

This study indicates that elite male and female soccer players have a similar risk for disordered eating, but neither have a higher risk for disordered eating than the general public. Future research should focus on clinical interviews to help better determine the extent of disordered eating in elite male and female soccer players.
